# 孤立性肺结节直径大小与临床及病理关系的初步研究

**DOI:** 10.3779/j.issn.1009-3419.2010.06.008

**Published:** 2010-06-20

**Authors:** 德松 杨, 运 李, 军 刘, 冠潮 姜, 剑锋 李, 辉 赵, 帆 杨, 彦国 刘, 足力 周, 梁 卜, 俊 王

**Affiliations:** 100044 北京，北京大学人民医院胸外科胸部微创中心 Department of Toracic Surgery, Peking University People's Hospital, Beijing 100044, China

**Keywords:** 孤立性肺结节, 直径, 临床表现, 病理, Solitary pulmonary nodules, Diameter, Clinical manifestation, Pathology

## Abstract

**背景与目的:**

孤立性肺结节（solitary pulmonary nodules, SPN）是临床诊治的难题之一，不同直径大小SPN的临床及病例特点可能各不相同。本研究旨在探讨SPN直径大小与临床及病理之间的关系。

**方法:**

收集2000年1月-2009年7月在北京大学人民医院胸外科经手术切除明确病理诊断的SPN患者共390例。其中男性212例，女性178例。平均年龄57.1岁；结节最大径平均2.02 cm。按SPN最大径进行分组，其中最大径≤0.5 cm 16例（A组），0.5 cm-1 cm 58例（B组），1 cm-2 cm 163例（C组），2 cm-3 cm 153例（D组），比较各组的临床表现及病理特点。

**结果:**

肿瘤剜除术20例，楔形切除153例，肺叶切除217例。术后病理：良性病变130例（33.3%），恶性病变260例（66.7%）。58.5%的SPN不伴有临床症状，随着结节直径增大，出现临床症状的比率有逐渐增高趋势。A、B、C、D四组的恶性比率分别为43.7%、50.0%、63.2%、79.1%，随着结节直径增大，恶性肿瘤机率显著增加，差异有统计学意义（χ^2^=22.535, *P* < 0.001）。单因素及多因素*Logistic*回归分析结果显示，SPN直径大小是良恶性判断的独立危险因素（OR=1.922, *P* < 0.001）。本组14例患者术前观察时间达到或超过2年，其中10例术后为恶性，观察期间无增大者7例，3例证实为恶性（42.9%）。

**结论:**

SPN直径大小与患者是否伴有临床症状相关，是病理性质判断的重要危险因素。临床发现的SPN应早期诊断，及时治疗。

## Abstract (en)

**Background and objective:**

Solitary pulmonary nodules (SPN) is a knotty problem in clinical work. The clinical manifestation and pathological features of SPN in sizes may be obviously different. The aim of this study is to analyze the relationship between the diameters and clinicopathologic characteristics of SPN.

**Methods:**

390 patients (212 male, 178 female, ranging from 17 years to 86 years, median age 57.1 years) with SPN who were postoperatively diagnosed by pathology between Jan. 2000 and Jun. 2009 are enrolled to this study. In these cases, the number of SPN diameters which are smaller than 0.5 cm (including 0.5 cm) (group A) is 16, between 0.5 cm and 1 cm (including 1 cm, not including 0.5 cm) (group B) is 58, between 1 cm and 2 cm (group C) is 163, and between 2 cm and 3 cm (group D) is 153. The clinical manifestation, and postoperative pathological characters are analyzed in various diameter ranges.

**Results:**

All procedures were carried out securely, including tumor enucleation (*n*=20), wedge resection (*n*=153), lobectomy (*n*=217). There are 130 benign cases (33.3%) and 260 malignant cases (66.7%). 58.5% SPN are detected incidently with no symptoms. There is an increasing incidence of clinical symptoms as the increasing diameter of SPN. All the cases are divided into 4 groups, the prevalence of malignant are separated 43.7% (group A), 50.0% (group B), 63.2% (group C), 79.1% (group D). The diameter of SPN is positively correlated with incidence of malignancy (χ^2^=22.535, *P* < 0.001). Single factor analysis and *Logistic* regression analysis show the diameter of SPN is an independent risk factor of malignant pathology (OR=1.922, *P* < 0.001). Fourteen patients in all of the SPN were followed up by 2 years or more before operation, and 10 of them turn out to be malignant at last. In the 14 patients, 7 cases were found not enlarged by CT scan, and 3 of them (42.9%) were malignant.

**Conclusion:**

The diameter of SPN is highly associated with clinical symptoms, and is a significant risk factor to predict pathology. Early diagnosis and treatment is so important for patients with SPN.

孤立性肺结节(solitary pulmonary nodules, SPN)是临床诊断难题，也是近年来研究的热点之一。不同直径大小的SPN，其临床表现及病理性质可能有所不同，研究不同直径大小的SPN特点对临床诊断和治疗决策意义重大。本研究旨在回顾性分析北京大学人民医院胸外科经手术明确病理诊断的SPN患者390例，比较不同大小SPN的临床特点及病理特征，具体如下：

## 1 临床资料及方法

### 1.1 临床资料

2000年1月-2009年7月在北京大学人民医院胸外科经手术切除明确病理诊断的孤立性肺结节患者共390例。其中男性212例，女性178例。年龄(57.1±14.8)岁(17岁-86岁)。结节最大径为(2.02±0.78)cm(0.2 cm-3 cm)。按SPN最大径分为4组，其中最大径≤0.5 cm16例(A组)、0.5 cm-1 cm 58例(B组)、1 cm-2 cm 163例(C组)、2 cm-3 cm 153例(D组)。

### 1.2 手术方法

全部患者均通过手术切除肺结节获得病理学诊断。手术方式包括：①肿瘤剜除术：电凝切开肿瘤浅层的肺组织至肿瘤表面，钝性将肿瘤剥除后放置于标本袋内经切口取出，肺组织断面以丝线间断水平褥式缝合或以内镜切开缝合器闭合；②肺楔形切除术：用器械固定肿瘤，用直线切开缝合器按“剥香蕉皮法”距离肿瘤1 cm的正常肺组织处将其完整切除，放置于标本袋内取出；③肺叶切除术：标准的解剖性肺叶切除，并根据快速冰冻病理结果同期进行系统性淋巴结清扫。

### 1.3 统计学方法

应用SPSS 13.0软件，计数资料行χ^2^检验，计量资料行*t*检验及单因素和多因素*Logistic*回归分析。以*P* < 0.05为差异有统计学意义。

## 2 结果

### 2.1 手术方式及病理类型

全组手术顺利。行肿瘤剜除术20例，楔形切除153例，肺叶切除217例。全组390例均获病理学诊断，术后病理：良性病变130例(33.3%)，包括结核瘤49例，错构瘤33例，炎性假瘤27例，硬化性血管瘤9例，霉菌球3例，肺囊肿2例，淋巴组织增生2例，支气管扩张2例，动静瘘1例，间质性肺炎1例，神经纤维瘤1例；恶性病变260例(66.7%)，包括腺癌164例，鳞癌40例，肺泡细胞癌23例，肺转移癌20例，小细胞癌9例，类癌4例。

### 2.2 SPN直径大小与临床表现的关系

全组中因查体或诊治其它疾病发现(简称“查体发现”)的患者为228例(58.5%)，其余162例患者因相关症状就诊而发现(41.5%)。A、B、C、D四组直径大小与临床表现的关系(表 1)，SPN直径越小，相关临床表现越少，而由查体发现的比率有增高趋势(χ^2^=3.612, *P*=0.307)。

**1:** SPN直径大小与临床表现的关系 Relationship between diameter and clinical manifestation of SPN

Diameter (cm)	Found by physical examination [*n*(%)]	Found by symptoms [*n*(%)]	Total (*n*)	*X*^2^	*P*
≤0.5	12 (75.0%)	4 (25.0%)	16	3.612	0.307
0.5-1	37 (63.8%)	21 (36.2%)	58		
1-2	96 (58.9%)	67(41.1%)	163		
2-3	83 (54.2%)	70 (45.8%)	153		
Total	228 (58.5%)	162 (41.5%)	390		

### 2.3 SPN直径大小与良恶性的关系

良性肿瘤大小平均为(1.71±0.78)cm，恶性肿瘤大小平均为(2.20±0.74)cm，差异有统计学意义(*Z*/*t*=-5.836, *P* < 0.001)。

A、B、C、D四组直径大小与良、恶性比率的关系(表 2)，SPN直径越大，恶性可能性越高(χ^2^=22.535, *P* < 0.001)。

**2:** SPN直径大小与良、恶性比率的关系 Relationship between diameter and pathology of SPN

Diameter (cm)	Benign [*n*(%)]	Malignant [*n*(%)]	Total (*n*)	*X*^2^	*P*
≤0.5	9 (56.3%)	7 (43.7%)	16	22.535	< 0.001
0.5-1	29 (50.0%)	29 (50.0%)	58		
1-2	60 (36.8%)	103 (63.2%)	163		
2-3	30 (20.9%)	121 (79.1%)	153		
Total	130 (33.3%)	260 (66.7%)	390		

### 2.4 SPN术前随诊2年以上者直径有无变化与病理结果的关系

全组术前观察达到或超过2年者14例，病理证实恶性者10例。14例中观察达到或超过2年无增大者7例，3例证实为恶性(42.9%)；有增大者7例均为恶性(100%)。

### 2.5 肿瘤良恶性相关危险因素分析

收集年龄、性别、病程、症状、吸烟史及吸烟量、既往肿瘤史、肿瘤家族史、影像学检查表现(肿瘤部位、肿瘤最大径、有无钙化、毛刺、分叶、胸膜牵拉征、界限清楚)及术中探查有无胸膜皱缩征等相关资料。单因素及多因素*Logistic*回归分析显示，患者年龄增加、肿瘤直径增大或出现胸膜皱缩，是提示恶性肿瘤的危险因素，而肿物边界清楚或出现钙化则是保护性因素(表 3，表 4，表 5)。

**3:** 临床资料与病变性质之间单因素分析 Single factor analysis between pathology and clinical data

	Benign (*n*=130)	Malignant (*n*=260)	*P*
Age (year)	48.4±14.7	61.6±11.3	< 0.001
Course of disease (month)	3.60±8.07	3.60±8.37	0.494
Diameter (cm)	1.71±0.78	2.20±0.74	< 0.001
Gender			0.314
Male	66 (50.8%)	146 (56.2%)	
Female	64 (49.2%)	114(43.8%)	
Symptoms			0.827
None	75 (57.7%)	153 (58.8%)	
Yes	55 (42.3%)	107(41.2%)	
History of smoking			0.078
Yes	43 (33.1%)	110(42.3%)	
None	87 (66.9%)	150 (57.7%)	
Quantity of smoking (smoking index: pack-years)	191.3±375.7	232.4±392.6	0.105
Calcification			0.001
None	113 (86.9%)	249 (95.5%)	
Have	17 (13.1%)	11 (4.2%)	
Spiculesign			< 0.001
None	112 (86.2%)	176 (67.7%)	
Have	18 (13.8%)	84 (32.3%)	
Signoflobulation			0.009
None	112 (86.2%)	194 (74.6%)	
Have	18(13.8%)	66 (25.4%)	
Traction of pleural			0.005
None	113 (86.9%)	194 (74.6%)	
Have	17(13.1%)	66 (25.4%)	
Circumscribed			< 0.001
No	66 (50.8%)	219 (84.2%)	
Yes	64 (49.2%)	41 (15.8%)	
Depression of pleural			< 0.001
No	121 (93.1%)	173 (66.5%)	
Yes	9 (6.9%)	87 (33.5%)	
Family history of tumor			0.142
No	122 (93.8%)	231 (88.8%)	
Yes	8 (6.2%)	29(11.2%)	
History of tumor			0.112
No	122 (93.8%)	235 (88.8%)	
Yes	8 (6.2%)	25(11.2%)	
Site Upper left	30(23.1%)	59 (22.7%)	0.400
Lower left	28(21.5%)	39(15.0%)	
Upper right	33 (25.4%)	86 (33.1%)	
Middle right	12 (9.2%)	25 (9.6%)	
Lower right	27 (20.8%)	51 (19.6%)	

**4:** 初步*Logistic*回归分析 Initial *Logistic* regression analysis between pathology and clinical data

	*P*	OR	95%CI
Age (year)	< 0.001	1.037	1.018-1.055
Diameter (cm)	< 0.001	1.885	1.342-2.650
Calcification	< 0.001	0.155	0.055-0.442
Spiculesign	0.250	1.529	0.742-3.152
Signoflobulation	0.124	1.856	0.844-4.082
Traction of pleural	0.577	0.811	0.388-1.695
Circumscribed	< 0.001	0.304	0.171-0.543
Depression of pleural	0.001	4.299	1.843-10.028

**5:** 进一步*Logistic*回归分析 Further *Logistic* regression analysis between pathology and clinical data

	*P*	OR	95%CI
Age (year)	< 0.001	1.040	1.022-1.058
Diameter (cm)	< 0.001	1.922	1.369-2.697
Calcification	0.002	0.202	0.073-0.558
Circumscribed	< 0.001	0.305	0.176-0.529
Depression of pleural	< 0.001	4.352	1.940-9.767

## 3 讨论

孤立性肺结节是指肺实质内直径不超过3 cm的单发圆形、类圆形实性病灶，不伴有纵隔淋巴结肿大、肺不张和周围浸润。SPN在临床上较为常见，Comstock等^[1]^报道正常人群普查发现孤立性肺结节的比率达0.2%。随着CT检查的普及，SPN在临床检查中发现的比率明显增加。文献^[1-3, 6-10]^报道SPN的恶性比率不尽相同，从5%到69%不等，本组结果显示，SPN的恶性比率为66.7%，且以腺癌为主，占63.1%(164/260)。

多数SPN患者是查体时偶然发现肺部结节，文献报道^[2-4]^无症状患者的比例为35%-90%。本组查体发现的SPN患者占全部患者的58.5%，与文献报道相符。分组比较发现，直径越小因查体发现的比率则逐渐增高，但*P*值为0.307，差异无统计学意义，分析其原因，可能与入组病例较少、存在统计学偏差有关。SPN早期出现相关临床症状者较少，定期体检是发现SPN的重要手段。

SPN良恶性的判定是临床诊治的难题，也是影响治疗选择的关键因素。通过单因素及逻辑回归分析显示，肿瘤直径增大、患者年龄增加或出现胸膜皱缩，均为提示恶性肿瘤的危险因素，而肿物边界清楚或出现钙化则是保护性因素。有关患者年龄、CT表现为肿物边界是否清楚或有无钙化及术中所见有无胸膜皱缩与SPN良恶性的关系文献报道较多，且结论一致^[2, 3, 5-7, 9-11]^，本文不予进一步论述。分析直径大小与SPN良恶性的关系可见，SPN直径大小是良恶性判断的独立危险因素(OR=1.922, *P* < 0.001)，且进一步分析SPN直径变化与其良恶性的关系可见，随直径的增大，恶性比率有明显增高趋势(χ^2^=22.535, *P* < 0.001)。从本组数据可见，直径超过2 cm的SPN，恶性可能性甚至接近80%，与文献报道相符^[11, 12]^，必须采取积极的态度去处理。但是更值得注意的是 < 5 mmSPN的处理态度，因为国外部分作者认为对于 < 4 mm结节没必要进一步随诊处理，认为其没有发生肺癌的风险^[13]^，但是本组≤5 mm的SPN恶性肿瘤的比例高达43.7%，远远高于文献报道^[11, 17]^。分析其原因，一方面是由于本组病例数尚少，另一方面本组是限于在外科接受治疗的患者，经过内科和放射科的筛选，所以可能存在一定统计学偏差，但是如此之高的恶性比例仍然提示我们，即使很小的肺内结节也需高度重视，不能断然拒绝手术治疗。

不仅某一时间点的结节大小对良恶性判断有影响，随诊期间结节大小的变化对良恶性的判断可能更有价值。美国胸科医师学会推荐考虑良性可能性大的SPN的患者的随诊时间为2年，认为如随诊2年结节大小未发生变化，则没有必要继续随诊^[6, 14]^。但是本组患者发现SPN后随诊超过2年的共14例，其中的7例随诊超过2年结节大小没有变化，3例经手术证实为恶性肿瘤(42.9%, 3/7)，而在2年中结节的直径增大的7例术后全部证实为恶性肿瘤。肺癌的发生经历增生-不典型增生-原位癌-浸润性癌等一系列阶段，其共同的规律是起初发展很慢，待发展到一定阶段速度会迅速增加，肺癌自细胞增生发展到1 cm大小约需经过10年-12年，而从1 cm发展到2 cm仅需2年时间^[15]^；肺泡细胞癌等进展缓慢的肿瘤，甚至可以在更长的时间不发生影像学上的明显变化。因此对于肺内小结节，特别是直径越小的结节，随访越应该慎重，不能因为短期内不发生变化就武断地将其归为良性病变而断然中止随访。

孤立性肺结节位于肺组织深部，难以取得病理活检标本，是临床诊治的难题之一。尤其对于长期随诊影像学表现无明显增大、临床趋于良性的肺内占位，创伤大的开胸手术使许多医师犹豫不决，从而失去早期治疗时机。肺癌是目前死亡率最高的恶性肿瘤，总体5年生存率低于15%，但早期肺癌(特别是Ⅰa期肺癌)经过合理治疗，其5年生存率可提高到80%以上^[16, 17]^。本文显示SPN中早期肺癌所占比率极高，如能通过常规体检、筛查肺部早期病变并予高度重视、早期干预，对提高肺癌治愈率、改善预后意义重大。而VATS是SPN较好的诊断兼治疗办法^[17-19]^。
